# Redesign of quality improvement work methods in the community in the health care organization in Israel

**DOI:** 10.1186/1472-6963-14-S2-P47

**Published:** 2014-07-07

**Authors:** Margalit Goldfracht, Mina Rotem, Silvia Fleishman, Calanit Key, Nicky Lieberman

**Affiliations:** 1 Clalit Health Services, Community Division, Tel-Aviv, Israel; 2 Family Medicine Department, Rappaport Medical School, Technion, Haifa, Israel

## Background

Comprehensive and coordinated management of chronic disease is a major challenge for healthcare systems. Clalit Health Services, a non-profit health fund, insures 4 million people in Israel, and provides comprehensive care through 1,300 primary and secondary clinics and 14 hospitals. The base of the health care is 1150 multi-professional primary care health centers. Clalit has a computerized database that tracks all services used by the members of the health fund. The data is linked to performance measurements by 52 indicators of care. This study describes the introduction and implementation of an innovative work model and assesses its influence on quality improvement, patient satisfaction, and waiting times, without the need for additional resources.

Redesign of the quality improvement actions in the clinic using the unique features of each profession to improve clinical quality and performance with emphasis on team work and use of the computerized indicators’ data for initiation of proactive interventions in patients’ care. Interventions included structured work process from population level to single patient level and computerized tools supporting the identification of patients for recall and active intervention during the visit. The only resources were reserved time for primary care providers to concentrate on clinical quality promotion. The adoption of the program was promoted by widespread publishing of the results in the organization and continuous follow up of the results.

## Materials and methods

The model was tested in 10 primary care clinics with matching controls in 2007-2008. The pilot group had 64,760 patients, and the control group had 65,759 patients. Performance of mammography was the common indicator in all the clinics. Waiting times for appointment, average cost per patient, and patient satisfaction were also measured according to Clalit’s biannual nationwide survey. The organization wide implementation was followed by intranet survey of all clinics biannually

## Results/Conclusions

One year after the intervention, mammography rates in the pilot group rose by 25.6% from 52.23% to 64.60% compared to the control group whose performance rate rose from 59.16% to 63.68%, an improvement rate of 8.1% (p=0.002). There was no worsening in outcomes of waiting times, costs, or patient satisfaction. 588 clinics’ managers answered to intranet survey in 2013, 40% response. 94% reported on implementation of the proactive model in the clinic. The general compound quality score of the organization rose 2%-5% each year since 2007.

